# PSYCHOLOGICAL WELL-BEING IN OLDER ADULTS WITH TREATMENT-RESISTANT DEPRESSION

**DOI:** 10.1093/geroni/igac059.1086

**Published:** 2022-12-20

**Authors:** Selmi Kallmi, Ann Steffen, Eric Lenze

**Affiliations:** University of Missouri-St. Louis, St. Louis, Missouri, United States; University of Missouri-St. Louis, St. Louis, Missouri, United States; Washington University School of Medicine in St. Louis, St. Louis, Missouri, United States

## Abstract

Geriatric psychiatry research has documented the importance of psychological well-being to older adults diagnosed with depression (Lenze et al., 2016). This presentation utilizes pre-treatment data from the Optimum Study of treatment-resistant depression in older adults recruited from five USA and Canadian metropolitan areas (N = 529). Social participation was measured with the PROMIS scale: Ability to Participate in Social Roles and Activities (Hahn et al., 2014). Positive affect and life satisfaction were assessed using scales from the Psychological Well-Being subdomain (Salsman et al., 2013) of the NIH Toolbox for Assessment of Neurological and Behavioral Function - Emotion Battery (NIHTB-EB). Along with associations between social participation and positive affect (r = .38, p < .01) and between social participation and life satisfaction (r = .26, p < .01), path analyses explored social participation as a mediator of the relationship between cognitive functioning (NIHTB-CB; Weintraub et al., 2013) and psychological well-being.

